# Aggressive Driving Behaviours in Cannabis Users. The Influence of Consumer Characteristics

**DOI:** 10.3390/ijerph18083911

**Published:** 2021-04-08

**Authors:** Sonia Ortiz-Peregrina, Carolina Ortiz, Rosario G. Anera

**Affiliations:** Laboratory of Vision Sciences and Applications, Department of Optics, University of Granada, 18071 Granada, Spain; soniaortiz@ugr.es (S.O.-P.); rganera@ugr.es (R.G.A.)

**Keywords:** aggressive driving, risk, driving safety, cannabis, THC, DUIC, visual quality, visual fitness-to-drive, DDDI

## Abstract

This study analysed dangerous driving behaviours in twenty young occasional cannabis users through objective and self-reported data, studying the relationship between the two aspects. Visual function was assessed in a baseline session and after smoking cannabis, as well as speed-related behaviour in a driving simulator. The participants responded to questionnaires on sociodemographic factors, their consumption profile, and the incidence of dangerous behaviours (Dula Dangerous Driving Index; DDDI). After cannabis use, the results revealed a significant deterioration in visual function. In terms of speed management, they showed significantly greater acceleration force in the two different sections of the route, and they drove significantly faster. Our correlations indicate that males and heavier users display more risky speed management. Likewise, the heavier cannabis users admitted to increased dangerous driving behaviour, and an accident in the preceding year was associated with a trend towards aggressive driving behaviour according to the DDDI questionnaire. The findings of this study suggest that cannabis users adopt dangerous behaviours when driving, despite the effect this drug has on certain important functions, such as vision. The results suggest a need for awareness-raising and information campaigns.

## 1. Introduction

Cannabis is the most widely consumed illicit drug in the world. According to the 2020 European Drug Report, it is the most widely used drug in Europe. Almost 8% of European adults have consumed cannabis in the past year, as have 15% of young adults (15–34 years) [1]. In Spain, data from the 2018 report of the National Institute of Toxicology and Forensic Sciences (INTCF) indicates that illegal drugs and psychopharmacological drugs were present in more than 43% of drivers who died in traffic accidents, with cannabis and cocaine being the most frequently detected illicit substances (60% and 51%, respectively).

Epidemiological studies have concluded that driving under the influence of cannabis, whose main psychoactive component is THC (∆^9^–tetrahydrocannabinol), can almost double the risk of an accident [2,3]. The same increment was estimated by a systematic review and meta-analysis, which added the special implication of cannabis in fatal crashes [4]. Likewise, other studies have indicated an association between cannabis use and traffic accidents [5], with this substance also increasing the probability of being at fault [6]. Past research has shown that acute intoxication can affect the driving skills needed for safe driving [7,8,9]. Hartman et al., demonstrated the effects on both longitudinal and lateral control, including higher lane weaving, decreased speeds, and higher headway following distances [10,11]. Likewise, one of our recent studies showed that occasional cannabis users drive outside the lane limits for longer and have a higher SDLP (standard deviation of lateral position) under acute intoxication [12].

The changes in driving performance reported in previous studies may be caused by the alterations that cannabis compounds generate in key functions needed for driving. It has been demonstrated that cannabis use affects aspects such as executive function (time and tasks management) [13,14,15], attention capacity [16], and processing speed [14]. Moreover, cannabis also seems to alter the main sensorial mechanism involved in driving, visual functioning [17]. It is estimated that vision constitutes the input channel for 90% of the sensory information used while driving [18]. Several studies have demonstrated slowed transmission of information through the visual path [19,20]. In functional terms, cannabis seems to alter important visual parameters, including visual acuity, contrast sensitivity, colour discrimination, ocular movements, and stereoscopic perception [12,17,21,22,23,24,25]. Previous evidence has shown that visually impaired drivers adopt some modifications in their driving behaviour as a strategy to compensate for their decreased visual capacity [26,27,28]. These modifications include avoiding driving on highways or reducing their speeds [26,27,28,29]. Similarly, the visual changes caused by cannabis use could make drivers feel less confident when driving, thus influencing their behaviour at the wheel. 

Cannabis users also seem to have a tendency to exhibit risky behaviours. The study of Vassallo et al., related driving under the influence of cannabis (DUIC) to speeding and other dangerous on-road behaviours [30]. Richer and Bergeron used self-reported and objective data, and found a link between DUIC and risky driving and negative emotional driving [31]. These authors observed a relationship between higher maximum speeds reached on a simulator and greater incidence of self-reported dangerous behaviours. Similarly, Bergeron and Paquette found that more frequent cannabis use and DUIC were associated with higher maximum speeds [32]. However, these findings do not agree with other studies on driving simulators which reported a decrease in mean speed when driving after cannabis use [8,10,33,34]. For other speed-related parameters, such as speed variability, few simulator-based studies have found changes after cannabis use [8,11,33,34]. 

The aim of this study is, therefore, to analyse the effect that smoking cannabis has on different driving performance parameters related to risk-taking behaviours (speed management variables), as well as on one of the key aspects for safe driving, visual function. Finally, we consider it interesting to analyse the relationship between objective driving performance data, visual effects after smoking cannabis, demographics, and self-reported data (i.e., consumption profile and self-reported dangerous behaviours when driving). 

## 2. Materials and Methods

### 2.1. Participants

The study included 20 volunteers (mean age 23.3 ± 4.4 years; range 19–36 years; five females), all of whom were occasional cannabis users, defined as self-reported cannabis consumption of ≥1 but <4 times/week over the past three months [10,11]. Participants indicated a similar frequency of use since they were consumers, avoiding including those with more intense use in the past. They also had to meet other inclusion criteria, such as being licensed drivers with at least one year’s experience, a driving frequency of at least once a week, at least 6/6 (Snellen notation) monocular visual acuity with their habitual correction worn for driving, and no binocular vision problems. 

To identify possible alcohol or cannabis use disorders, we employed the Alcohol Disorders Identification Test (AUDIT; cut-off score >12 for women and > 14 for men) [35] and the Cannabis Use Disorders Identification Test-Revised (CUDIT-R; cut-off score >12 points) [36]. If the volunteers exhibited a use disorder for either of these substances they were excluded, as well as if they used other drugs, were pregnant or breastfeeding in the case of females, and if they suffered simulator sickness. 

This study was in line with the Declaration of Helsinki and was prospectively approved by the University of Granada Human Research Ethics Committee (921/CCEIH/2019). Before the testing sessions, the volunteers were interviewed and informed of the procedures, details and possible consequences of this study, and they gave their informed consent. 

### 2.2. Visual Function 

Visual function was evaluated binocularly (the normal state for driving) with the habitual optical correction employed for driving (if any). For this purpose, we applied three visual tests: visual acuity, contrast sensitivity, and stereoacuity. The first two parameters and stereoacuity for far distance were evaluated using the charts available in the POLA VistaVision Visual Chart System (DMD Med Tech srl. Torino, Italy). Visual acuity, or the capacity to resolve high-contrast stimuli in detail, is the standardised test employed by the licensing authorities [37]. It was measured at 5.5 m and the results were expressed on a logMAR scale. Contrast sensitivity refers to the capacity of the visual system to distinguish not only size, but also contrast. Although this visual function is not commonly included in drivers’ visual exams [37], its importance has been highlighted by the literature on vision and driving [38,39,40]. We obtained the minimum contrast required to see a visual grating target over a uniform background, termed the contrast threshold. Contrast sensitivity is the inverse of the contrast threshold for a given spatial frequency. The spatial frequencies tested were 0.75, 1.5, 3, 6, 12 and 18 cycles per degree (cpd). Each of these had 8 contrast levels and were evaluated at the recommended distance of 2.5 m. 

Stereoacuity refers to three-dimensional vision and is defined as the capacity to distinguish the spatial location of objects in the environment. This visual function is non-standardised [37] in drivers’ visual exams, despite the importance of accurate spatial orientation and localisation when driving. For far-vision (5.5 m), we employed the chart in the system mentioned above. The stereotest can measure disparities ranging from 300 to 10 arcsecs using rows of five polarised vertical lines at each level of disparity assessed. For near-vision testing, we employed the Fly Stereo Acuity Test with LEA Symbols (Stereo Optical Co., Inc., Chicago, IL, USA). This is a polarised-based test that contains a graded circle test, assessing disparities from 400 to 20 arcsecs. Stereoacuity was assessed while the subjects were wearing polarised glasses. 

### 2.3. Driving Simulator 

**Figure 1:**
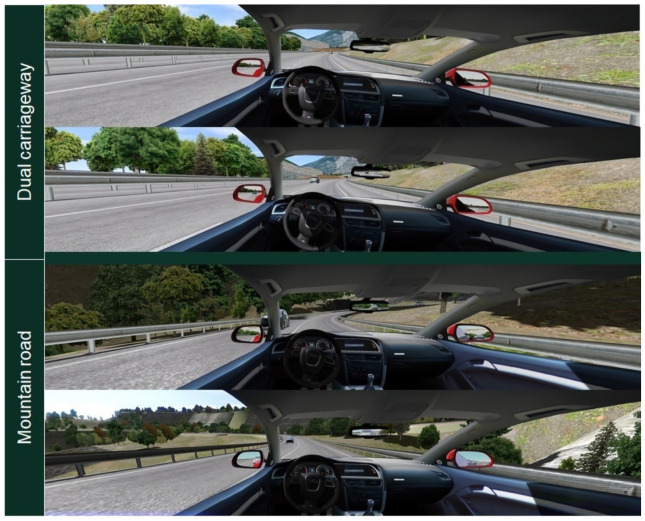
Screenshots of the main sections driven along the route: dual carriageway (two top images), and mountain road (two bottom images).

The study employed the SIMAX DRIVING SIMULATOR v.4.0.8 BETA (SimaxVirt S.L., Pamplona, Spain). This driving simulator, successfully used in previous studies [41,42,43], is fixed-based and provides a 180° field of view. The route comprised two main sections (dual-carriageway and mountain road) which totalled 10.5 km. A full description of the route sections is included in Table 1. The driving scenario was performed in daylight and under good weather conditions, as can be seen in Figure 1, which shows screenshots pertaining to the sections of the route. 

To objectively assess dangerous/risky driving behaviours in the simulator, we considered several variables related to speed management for each section: the maximum acceleration force and the maximum braking force (ranging between 0 and 1, where 0 is no pressure and 1 is the maximum pressure), the maximum speed reached along the route (km/h), and the speed adaptation (i.e., speed—speed limit, so that negatives values indicate driving under the speed limit) (km/h). 

### 2.4. Self-Reported Data: Demographics and DDDI Questionnaire

As part of the experiment, the participants completed several questionnaires. All of them were administered at the initial visit, ensuring that participants were not under the influence of cannabis or alcohol. Instructions for completion were administered verbally and were also written on each questionnaire. Subjects were instructed to respond based on their experience and took as much time as they needed. A member of the research team accompanied them and resolved any doubts about the content or meaning that arose. Participants completed the first questionnaire on socio-demographic characteristics including age, sex and driving experience. Furthermore, they completed another questionnaire on their cannabis and alcohol use, the frequency of driving under the influence of the two drugs, and self-perceived risk during DUIC. This questionnaire comprised 24 items based on a questionnaire from a previous study [44].

The Dula Dangerous Driving Index (DDDI) was employed to characterise aggressive behaviour among the participants [45]. It has been shown that this index can be used as a composite measure for dangerous driving, being valid for clinical use [46]. This is a 28-item questionnaire, where participants rate the frequency with which they exhibit certain behaviour, using a 5-point Likert scale ranging from 1 (never) to 5 (always). The total score can therefore range from 28 to 140. This questionnaire is comprised of three subscales: “risky driving” (RD, 12 items); negative cognitive/emotional driving (NCED, nine items); and “aggressive driving” (AD, seven items). The mean score and total score were obtained for each subscale as well as the total DDDI score, by summing either the score for each item in the subscale or the whole questionnaire, respectively. 

### 2.5. Procedure

Once the participants had been informed about the study and had given their informed consent, they underwent two training sessions with the driving simulator in order to familiarise them with the system. The training sessions lasted about 15 min and involved a similar scenario to those used in the experimental drives, but with no traffic or pedestrians. Participants took a washout period of one week between training sessions, in order to avoid any possible learning effects. If any symptoms of simulator sickness were noted, the session was interrupted and the participant was excluded from the study.

When the training sessions had been completed, the two experimental sessions (one involving no substance use and another after smoking a cannabis cigarette) were conducted in a random order and at least one week apart. Each participant prepared the cannabis cigarette in the way they usually do for their habitual consumption, given that our aim was to simulate real use. They smoked the cigarette in about 10 min, with no particular procedure being imposed, thus simulating their normal consumption behaviour. In each session, which lasted approximately 75 min, the participants did the visual tests and drove the route in the simulator, also in a random order. This duration guarantees a considerable psychoactive effect during the session, given that this effect tapers off within 2–3 h [47]. As a safety precaution, the participants committed to not driving to and from the laboratory. Moreover, they were all asked to abstain from alcohol for the 24 h prior to each testing session and from cannabis use for four days. To obtain an objective drug-intake screening during the sessions, a saliva drug test (Dräger DrugTest 5000, Dräger Safety AG & Co. KGaA. Lübeck, Germany) was performed, which detects cannabis, amphetamines, benzodiazepines, cocaine, methamphetamines, opiates, methadone, and ketamine. This drug test has been proposed as a sensitive and valid method for detecting cannabinoids in oral fluid [48,49]. For alcohol screening, we measured breath alcohol content (BrAC) with a Dräger Alcotest 7110 MK-III. 

### 2.6. Data Analysis

All the statistical procedures were performed using SPSS 26.0 software (SPSS Inc., Chicago, IL, USA). Descriptive statistics, including means and standard deviations (SDs) were obtained for sociodemographic, vision, and driving variables. The frequency distribution was also included to describe the results from the self-reported questionnaires. Comparisons between testing conditions (baseline and after smoking cannabis) were made through the paired *t*-test and Wilcoxon test, depending on the data distribution (Kolmogorov-Smirnov test).

To obtain a general score index quantifying the overall change experienced in visual function after cannabis use, we calculated the Overall Visual Change Index. For each visual parameter, we found the change after cannabis use (i.e., the value from the baseline session minus the value after smoking cannabis). Then, z-scores for the change in each parameter were computed, transforming the variables wherever necessary so that a negative value indicated greater deterioration than the mean. Then, a mean z-score considering all visual parameters was obtained, the value of which constituted the Overall Visual Change Index. 

As an objective quantification of the driving style, we also computed the Speeding Index. This index was obtained as in previous works for computing overall driving performance scores [12,43,50], by calculating the z-scores for each driving variable and averaging these to obtain a general index for each participant. On this occasion, a positive value for all of them indicated a more aggressive driving style or greater risk while driving. The Speeding Index was calculated for both sessions (baseline and after smoking cannabis), and the two conditions were also averaged to provide a general Speeding Index that could be used to correlate with other variables. 

Both indexes were employed to assess the relationship between self-reported data, driving behaviour, and visual changes after smoking cannabis. In this way, we computed a correlation analysis (Spearman correlations) to investigate whether personal characteristics, driving style, or use profile could be associated with aggressive behaviour or an increased risk of driving under the influence of cannabis.

## 3. Results

All the participants stated that they drive at least once per week, with a mean of 4.5 ± 2.3 days per week. Their self-reported mean driving experience was 4.1 ± 4.0 years (range 1–17 years). The participants’ mean age when they first used cannabis was 17.6 ± 1.8 years. For the last six months, their self-reported cannabis-use frequency was: 30% 2–3 days/week; 10% once a week; 10% twice a month; 30% once a month; and 20% less frequently. The AUDIT questionnaire score was 7.1 ± 3.8 (max. score 40 points), and 6.1 ± 4.1 for the CUDIT-R (max. score 32 points). Finally, when subjects were asked if they felt their driving could be impaired after smoking cannabis, the response frequencies were: 5% “does not get worse”, 65% “gets slightly worse”, and 30% “gets much worse”.

### 3.1. Visual Assessment

**Figure 2:**
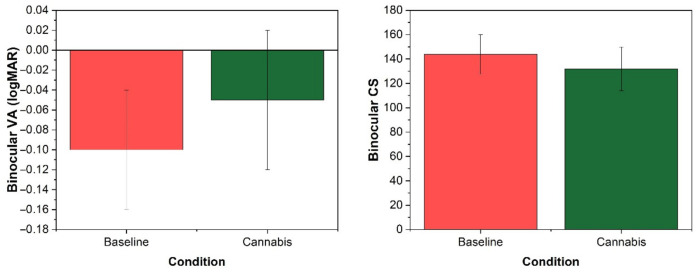
Change in visual acuity and contrast sensitivity after smoking cannabis.

**Figure 3:**
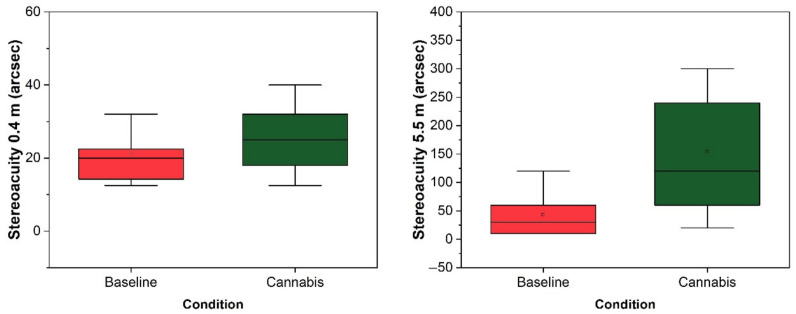
Change in stereoacuity after smoking cannabis for the two distances tested.

After smoking cannabis, the participants demonstrated a significant deterioration in visual function. Visual acuity demonstrated an average difference of 0.05 ± 0.05 logMAR units, being significantly worse after cannabis use (t = −4.516; *p* < 0.001) (Figure 2). Contrast sensitivity also worsened, with a mean reduction between conditions of 11.92 ± 16.54 (t = 3.223; *p* = 0.004) (Figure 2). Finally, stereoacuity was also altered, increasing an average of 111.05 ± 88.12 arcsecs for the farthest distance evaluated (5.5 m) (Z = −3.623; *p* < 0.001) and 10.50 ± 26.92 for the nearest distance (Z = −2.496; *p* = 0.013) (Figure 3). 

### 3.2. Risky Driving: Speed Management

Table 2 summarises the results of the variables analysed from the driving simulator with regard to the speed management. As can be seen, we found a significantly higher maximum acceleration force after smoking cannabis than in the baseline session, both on the dual carriageway and the mountain road. The maximum speed was higher than the speed limit for both conditions and on both sections of the route. However, average speed adaptation indicated that, most of the time and along the entire route, the participants drove under the speed limit (negative value), both in the baseline session and after smoking cannabis. Nevertheless, the participants drove faster after smoking cannabis than in the baseline session, with this difference being statistically significant for the mountain road section. 

### 3.3. Driving under the Influence of Cannabis (DUIC): Self-Perceived Risk and Behaviour

#### 3.3.1. General Profile of Use and Perception of Risk in Driving

All the participants were given the questionnaire used by Sexton et al., relating to their cannabis and alcohol use profile (as these are often related) [44]. All of them indicated that they smoked this substance in the company of other people (“social smokers”), while 5% also smoked alone. Three participants (15%) indicated that they had had a car accident in the preceding year, none of which involved injury to people. With regard to driving under the influence of alcohol, more than half of the participants (65%) indicated that they had done so when they were slightly intoxicated (after drinking less than four units of alcohol): 15% weekly; 30% monthly; 5% 2–10 times a year; 5% once a year; 10% once in their life. Surprisingly, a huge proportion of them (52.8%) had also done so after a higher alcohol intake (more than four alcohol units): 5.3% weekly; 21.1% 2–10 times a year; 5.3% once a year; 21.1% once in their life. For cannabis, the results were similar, 55% of the total sample reported driving under the influence of cannabis: 5% weekly; 5% monthly; 25% 2–10 times a year; 20% once in their life. Worryingly, 15% reported they had even smoked cannabis while driving. As for the combination of alcohol and cannabis, 25% said they had driven after using both substances: 5% weekly; 5% monthly; 5% once a year; 10% once in their life. On these occasions, 100% of the participants reported consuming less than four units of alcohol. 

All the participants were of the opinion that consuming more than four units of alcohol negatively affected their driving; 55% said that it “gets a little worse”, while 45% said that it “gets a lot worse”. Despite this, 52.8% acknowledged driving under these circumstances. For cannabis, the perception of risk is lower, with 5% saying their driving is “not worse after using cannabis”, while 65% think it is “a little worse” and only 30% consider it to be “a lot worse”. Furthermore, these last two groups believe that after consumption, their driving remains negatively affected for an average of 2 h. During cannabis intoxication, they claim to experience slower reaction speeds (79%), concentration and attention problems (63.3%), sleepiness (58.1%), fatigue (15.8%), vision problems (10.6%), sensitivity to light (5.3%), colour vision problems (5.3%), poor coordination (5.3%), and aggressive behaviour (5.3%).

#### 3.3.2. Dula Dangerous Driving Index

The item with the highest score in the DDDI questionnaire was *“I consider the actions of other drivers to be inappropriate or ‘stupid’*” (2.85 ± 0.67), corresponding to the *NCE (Negative Cognitive/Emotional Driving)* subscale. On the other hand, the item with the lowest score corresponded to the RD (Risky Driving) subscale: “*I ‘drag race’ other drivers at stop lights to get out in front*” (1.05 ± 0.22).

If we analyse by subscales, the most frequent negative feeling for the NCE was “*I consider the actions of other drivers to be inappropriate or ‘stupid’*.” The least frequent item was “*Passengers in my car/truck tell me to calm down*”. For the subscale Aggressive Driving (AD), the most frequent behaviours were “*I verbally insult drivers who annoy me*” and “*When I get stuck in a traffic jam I get very irritated*.” The lowest-scoring behaviour in this subscale was “*I deliberately use my car/truck to block drivers who tailgate me*”. Finally, for the subscale Risky Driving (RD), the most frequent behaviour was “*I will weave in and out of slower traffic*” while the least frequent was “*I will drive on the shoulder or median strip to get around a traffic jam.*” The mean score per subscale was highest for NCE (2.08 ± 0.59), followed by RD (1.55 ± 0.66) and, finally, AD (1.42 ± 0.36). This indicates that there is a higher incidence of negative thoughts or feelings while driving than actual risky or aggressive behaviour (Table 3).

#### 3.3.3. Correlations between Visual Outcomes, Speed Management, and Self-Reported Data

Table 4 shows the Spearman’s correlations obtained between all the aspects studied. The results show that frequency of consumption was significantly associated with the Speeding Index (ρ = 0.649; *p* = 0.003), indicating that heavier cannabis users engaged more in dangerous behaviours in terms of speed management on the simulator. Gender was significantly associated with driving speed, with men adopting riskier behaviour on the simulator, and having higher Speeding Indexes (ρ = −0.502; *p* = 0.029). Frequency of consumption was also associated with gender, being higher in men than in women (ρ = −0.509; *p* = 0.022). Confirming this finding, being male was also significantly associated with a higher score on the CUDIT-r test (ρ = −0.047; *p* = 0.048). In addition, those who drive the most are those who show the highest frequency of driving under the influence of cannabis (DUIC) (ρ = 0.481; *p* = 0.032).

It should be noted that a higher monthly consumption of cannabis significantly correlated with higher total scores in the DDDI (ρ = 0.591; *p* = 0.006) and in particular, with the Negative Cognitive/Emotional Driving subscale (ρ = 0.591; *p* = 0.006). In addition, a higher score in the AUDIT test significantly correlated with a higher score on the Risky Driving subscale (ρ = 0.670; *p* = 0.001), indicating that people who report more hazardous alcohol use also adopt risky behaviour behind the wheel more often. Similarly, the CUDIT-R score showed a significant correlation with the overall DDDI score (ρ = 0.674; *p* = 0.001), indicating that subjects with a more hazardous cannabis use adopt a more dangerous driving behaviour. Specifically, those with higher CUDIT-r scores, more frequently adopted the dangerous behaviours encompassed within Negative Cognitive/Emotional Driving (ρ = 0.524; *p* = 0.018) and Risky Driving, (ρ = 0.476; *p* = 0.034). Finally, it is important to note that the number of self-reported accidents significantly correlated with the DDDI Aggressive Driving subscale score (ρ = 0.469; *p* = 0.037), meaning this kind of driving behaviour could be the most dangerous. Finally, it is important to note that the deterioration in visual function after smoking cannabis was not associated with either the speed that drivers adopted or the self-reported data.

## 4. Discussion

Visual performance deteriorated significantly after cannabis consumption. Visual acuity, the visual function most widely employed to assess visual fitness-to-drive, was significantly impaired under the influence of cannabis. These results are in line with previous findings that suggest blurred vision in cannabis users [17,51]. In contrast to this, previous studies have failed to find impaired static visual acuity after cannabis use [52], although it has been found for dynamic visual acuity [53]. Contrast sensitivity, a key visual function for driving performance according to the literature on vision and driving [38,54], was also impaired. This result agrees with previous studies of acute intoxication [12,17], and those evaluating permanent changes in cannabis users [24,25]. With regard to three-dimensional perception, it has been demonstrated that stereoacuity is significantly impaired, meaning that drivers under the influence of cannabis could have spatial orientation and localisation problems [12,17]. Moreover, this result is in line with studies concluding that there is reduced occurrence of the binocular depth inversion illusion [55,56]. This optical illusion appears to change the three-dimensional aspect of an object if it is not congruent with the observer’s previous experience of that object [57]. Despite the significantly impaired visual function after smoking cannabis, the overall visual change index did not correlate with speed management or self-reported data. This could indicate that drivers are not sufficiently aware of the visual effects caused by cannabis use.

Our earlier research analysing the influence of this drug on a wide range of visual parameters and its relationship with self-perceived effects, indicated that only contrast sensitivity was significantly associated with the subjective perception of visual status following cannabis use [17]. Our index included this visual parameter, in addition to others, which could have resulted in the lack of a relationship with the driving data or other factors. Moreover, although cannabis users may perceive some visual effects, it is possible that the perceived change in visual quality is not enough to trigger a sense of risk that would activate a series of strategies to promote safety.

Thus, while we expected that drivers under the influence of cannabis would adopt a more cautious driving style due to the negative effect found on visual performance, an essential sensory mechanism for driving, we actually found a contrary tendency. Both on the dual carriageway and the mountain road, drivers pressed the accelerator significantly harder. On the mountain road, they also drove significantly faster than in the baseline session with respect to the speed limit, although they did not exceed it. A similar tendency was observed on the dual carriageway, but they drove nearer to the speed limit. On the dual carriageway, we found greater variability between the participants’ behaviour with regard to speed adaptation than on the mountain road, with some of them even driving above the speed limit. This result could be due to the fact that drivers may feel the dual carriageway is less dangerous/difficult than the mountain road. Nevertheless, the changes observed suggest a more dangerous driving style when driving under the effects of cannabis. In previous studies, the most widely analysed speed-related parameter is mean speed, and findings show that, in general, this decreases after cannabis use [8]. For example, the study by Hartman et al., carried out on 19 occasional cannabis users (using the same definition of occasional use as in this study) reported that cannabis use was associated with a reduction in mean speed and an increased tendency to drive below the speed limit in the driving simulator [11]. Also, Brands et al. found a reduction in speed in a driving simulator after cannabis use in 91 young occasional cannabis users [58]. Given this previous evidence and our own results, it seems important to analyse other speed-management parameters. However, other work does suggest a link between DUIC and higher maximum speeds, more in line with our results [31,32]. The speed-related behaviour found in our study could be caused by the over-confidence of our participants in their own capabilities when they are under the effect of cannabis, something that could also be concluded from their responses to the questionnaire. Our results reflect the fact that 65% of participants reported driving after drinking small quantities of alcohol (less than four units), and 52.5% had driven even after drinking heavily (more than four units). However, 100% of the participants thought that alcohol negatively affected their driving. Interestingly, the data reveals that the perception of risk associated with cannabis use while driving is lower, as although 55% of participants have driven after cannabis use, 5% think that their driving is not worse after smoking cannabis and 65% think that it is only slightly worse. In Spain, the DUIC percentage seems to be higher than in other countries, such as the United States or Canada, although in the latter it has been found that it is more common among young people to drive under the influence of cannabis than to drive after drinking alcohol [59,60].

The correlation analysis showed no relationship between the change in visual performance and participant’s driving behaviour. Vision is unarguably essential for driving [38], and previous research has demonstrated that visually impaired drivers adopt some strategies when driving trying to compensate for such impairment. For example, older drivers with cataract tend to avoid driving during rush hour, at night or in heavy traffic [27,28,61]. Likewise, these drivers tend to adopt lower speeds than older drivers without visual impairment [29,50]. The impairment of visual function caused by cannabis use may be not enough for the participants to perceive themselves as having visual difficulties, so it does not bear an influence on their risk management while driving. On the contrary, some studies have found a link between DUIC and dangerous driving behaviour in everyday life [32]. In general, the average scores reported for each DDDI subscale in our study are lower than those found by other authors [62,63]. This difference may be due to the personal traits of the participants, for example, agreeableness, which has been found to negatively correlate with the incidence of all types of dangerous behaviour [63]. Nonetheless, our correlation analysis of self-reported data showed some interesting associations. As mentioned, our results confirm heavier cannabis use in males [64], and being a male also correlated with a greater incidence of speed-related dangerous behaviours (higher speeding indexes) [65,66]. Moreover, the correlations showed that people with increased alcohol and cannabis use adopt a more dangerous driving style. Specifically, heavier cannabis users have higher speeding indexes and more negative emotional behaviour, and heavier cannabis and alcohol users were more vulnerable to risky driving practices. These results agree with other studies, also employing the DDDI [31,32], showing that DUIC is associated with more risky driving [60,67]. In the study by Bergeron and Paquette, the authors found no link between increased risky behaviour and traffic accidents [32]. However, in our study, the Aggressive Driving subscale score did correlate positively with the number of self-reported accidents in the previous year. A recent study employing the DDDI concluded that negativity biases could explain differences in dangerous driving and accident risk between drivers [68]. Our results also suggest that some dangerous behaviour is more closely linked to traffic accidents than others.

### Limitations of the Study

This study is not without limitations, and we must consider these when interpreting the results. Firstly, the use of a driving simulator supposes an important limitation due to its inability to completely represent the realism of the driving environment. The simulator is designed to reproduce the characteristics of the different environments as accurately as possible, but it cannot capture the dynamism of real-life driving. Nevertheless, this simulator has been used successfully in previous studies [41,43] and there is evidence to support the relative validity of driving simulators with respect to actual driving [69,70]. Simulators mean work can be conducted under safe conditions and in fully controlled situations, which would be impossible in real-world scenarios, and they are hugely important in studies like this one for ethical reasons. However, it is known that driving simulators are more sensitive to the effects of cannabis than on-road studies of driving performance [9,71], so the results obtained in simulator-based studies should be interpreted with caution when generalising them to a real situation.

Secondly, the fact that each participant consumed the THC cigarette as they usually do, does not allow us to establish a relationship between dose and effect. We might think that by not measuring blood concentration we are also unable to quantify the effect on vision and driving; however, the relationship between dose, blood concentration, and effect is non-linear [47,72]. Our goal was to study the consequences that a participant’s typical use, on a normal day, has on vision and driving, and thus be able to relate these two aspects from a real perspective. These results should serve as a starting point for future work involving varying doses, or different administration routes. Finally, it is important to consider that the limited sample size prevents the generalization of these results. Furthermore, the age and gender distribution does not represent the general population, as only young participants and a higher proportion of males were included. Although a lower incidence of cannabis-impaired driving has been demonstrated in older and female users [73,74,75], they might show different behaviours, so this issue should be considered in future studies. Therefore, further research with larger samples, different ages and similar gender distribution is needed to confirm the findings of this study.

## 5. Conclusions

Cannabis is one of the most commonly detected illicit drugs among drivers. This drug causes physical or cognitive alterations in functions needed for safe driving. In addition, previous research has demonstrated that cannabis users may be more prone to some dangerous driving behaviours. In this study, we analysed the effects of smoking cannabis on visual function and on several driving performance parameters related to risky behaviours. We also investigated dangerous driving behaviours using self-reported data. Finally, the relationship between all these aspects was analysed.

Despite the fact that vision, a key sensorial mechanism for driving, was significantly altered after cannabis consumption, the participants did not exhibit a more cautious driving style under these circumstances. In fact, some of the variables studied in this work, such as the acceleration pedal force and the speed relative to the speed limit, indicated a riskier attitude under the effects of cannabis. These results seem to suggest that cognitive changes due to cannabis use have a greater influence on driving behaviour than the sensory changes found in visual function. Also, being male and a heavier cannabis user correlated significantly with greater speeding indexes, suggesting a more dangerous or riskier driving style.

On the other hand, although our sample has lower scores in the different subscales of the Dula Dangerous Driving Index (DDDI), data on driver demographics and use profiles have revealed significant associations between increased cannabis use and dangerous driving behaviour. Moreover, our results suggest a lack of awareness of the risks associated with cannabis use in driving, given that a considerable proportion of participants have driven after smoking cannabis, and most think that it only slightly affects their ability to drive.

There is, therefore, still a considerable need for awareness-raising and information campaigns aimed at the general public, as well as for research that provides adequate insights into how this drug affects both short- and long-term vision and the ability to drive safely. Finally, testing positive for cannabis could alert the authorities about certain behaviours that may compromise safe driving.

## Figures and Tables

**Table 1:** Description of the different sections of the route.

	Speed Limit (km/h)	Length (km)	Other Traffic and Road Users	Road Configuration/Layout
Dual carriageway	120	4.5	Moderate Same direction	Two lanes in each direction Straight and gentle curves
Mountain road	40/90	6	Moderate Oncoming and same direction	One single lane carriageway Straight, slight bends, sharp bends, ascending and descending slopes

**Table 2:** Comparison of speed management variables in the baseline session and after smoking cannabis. Mean, SD, and the results of the statistical tests are included.

		Baseline Condition (Mean (SD)/Median (IQR))	THC Condition Mean (SD)/Median (IQR))	t/Z	*p*-Value
Dual Carriageway	Max acceleration force *	0.72 (0.62–1.00)	0.89 (0.71–1.00)	−2.179	0.029
	Max braking force *	0.17 (0–0.41)	0.02 (0–0.75)	−0.974	0.330
	Max speed (km/h) *	130.52 (126.10–138.36)	130.69 (127.26–138.40)	−0.597	0.550
	Speed adaptation (km/h)	−4.13 (6.60)	−1.73 (10.98)	−1.235	0.232
Mountain Road	Max acceleration force *	0.96 (0.80–1.00)	1.00 (1.00–1.00)	−2.314	0.021
	Max braking force *	1.00 (1.00–1.00)	1.00 (1.00–1.00)	−1.000	0.317
	Max speed (km/h)	105.58 (8.15)	103.20 (6.62)	1.123	0.275
	Speed adaptation (km/h)	−17.20 (2.15)	−15.91 (1.95)	−2.142	0.045
Total circuit	Speeding Index	−0.18 (0.40)	0.11 (0.43)	−2.831	0.011

* Median (IQR) and Wilcoxon Test results are shown.

**Table 3:** Scores obtained in the Dula Dangerous Driving Index Questionnaire (DDDI). For each item, the percentages according to frequency and the average score (SD) are indicated. For each subscale, the total average score ± SD is shown.

Dula Dangerous Driving Index (DDDI)		1 = Never	2	3	4	5 = Always	Mean (SD)
NCE1	I drive when I am angry or upset.	30%	15%	20%	15%	20%	2.80 (1.54)
NCE2	I lose my temper when driving.	60%	35%	5%	0%	0%	1.45 (0.61)
NCE3	I consider the actions of other drivers to be inappropriate or “stupid”.	5%	15%	70%	10%	0%	2.85 (0.67)
AD4	I flash my headlights when I am annoyed by another driver.	85%	10%	0%	5%	0%	1.25 (0.72)
AD5	I make rude gestures (e.g., giving “the finger”; yelling curse words) toward drivers who annoy me.	75%	25%	0%	0%	0%	1.25 (0.44)
AD6	I verbally insult drivers who annoy me.	45%	30%	15%	5%	5%	1.95 (1.15)
AD7	I deliberately use my car/truck to block drivers who tailgate me.	80%	15%	5%	0%	0%	1.25 (0.55)
AD8	I would tailgate a driver who annoys me.	95%	0%	5%	0%	0%	1.10 (0.45)
RD9	I “drag race” other drivers at stop lights to get out in front.	95%	5%	0%	0%	0%	1.05 (0.22)
RD10	I will illegally pass a car/truck that is going too slowly.	30%	45%	25%	0%	0%	1.95 (0.76)
AD11	I feel it is my right to strike back in some way, if I feel another driver has been aggressive toward me.	30%	50%	15%	5%	0%	1.95 (0.83)
NCE12	When I get stuck in a traffic jam, I get very irritated.	45%	35%	10%	10%	0%	1.85 (0.99)
RD13	I will race a slow-moving train to a railroad crossing.	95%	0%	5%	0%	0%	1.10 (0.45)
RD14	I will weave in and out of slower traffic.	25%	40%	20%	10%	5%	2.30 (1.13)
RD15	I will drive if I am only mildly intoxicated or buzzed.	45%	35%	20%	0%	0%	1.75 (0.79)
AD16	When someone cuts me off, I feel I should punish him/her.	90%	0%	10%	0%	0%	1.20 (0.62)
NCE17	I get impatient and/or upset when I fall behind schedule when I am driving.	10%	45%	35%	5%	5%	2.50 (0.95)
NCE18	Passengers in my car/truck tell me to calm down.	80%	20%	0%	0%	0%	1.20 (0.41)
NCE19	I get irritated when a car/truck in front of me slows down for no reason.	15%	45%	25%	10%	5%	2.45 (1.10)
RD20	I will cross double yellow lines to see if I can pass a slow-moving car/truck.	50%	40%	10%	0%	0%	1.60 (0.68)
RD21	I feel it is my right to get where I need to go as quickly as possible.	65%	25%	10%	0%	0%	1.45 (0.69)
NCE22	I feel that passive drivers should learn how to drive or stay home.	45%	40%	5%	5%	5%	1.85 (1.01)
RD23	I will drive on the shoulder or median strip to get around a traffic jam.	100%	0%	0%	0%	0%	1.00 (0.00)
RD24	When passing a car/truck on a 2-lane road, I will barely miss on-coming cars.	70%	20%	10%	0%	0%	1.40 (0.68)
RD25	I will drive when I am drunk.	70%	25%	0%	5%	0%	1.40 (0.75)
NCE26	I feel that I may lose my temper if I have to confront another driver.	45%	40%	10%	5%	0%	1.75 (0.85)
RD27	I consider myself to be a risk-taker.	40%	25%	25%	5%	5%	2.10 (1.17)
RD28	I feel that most traffic “laws” could be considered suggestions.	50%	45%	5%	0%	0%	1.55 (0.61)
	TOTAL SCORE	47.70 (10.04)					
	NCE SCORE (Negative Cognitive/Emotional Driving)	14.30 (3.92)					
	AD SCORE (Aggressive Driving)	9.95 (3.10)					
	RD SCORE (Risky Driving)	19.05 (3.53)					

**Table 4:** Spearman’s correlation coefficients found for self-reported data.

		1	2	3	4	5	6	7	8	9	10	11	12	13	14	15
1	*Age*	-														
2	*Gender*	−0.113	-													
3	*Cannabis use (monthly)*	−0.214	−0.509 *	-												
4	*Years driving*	0.909 **	−0.020	−0.242	-											
5	*Driving frequency (monthly)*	−0.188	0.138	0.029	−0.219	-										
6	*Frequency* *of DUIC*	0.052	−0.213	0.559 *	0.060	0.481 *										
7	*AUDIT*	−0.177	−0.071	0.332	−0.038	−0.459 *	−0.250	-								
8	*CUDIT-R*	0.054	−0.447 *	0.735 **	0.001	−0.233	0.295	0.549 *	-							
9	*Accidents (past year)*	0.099	−0.243	0.101	0.122	−0.219	−0.142	0.135	0.012	-						
10	*DDDI score*	−0.074	−0.241	0.591 **	−0.063	−0.199	−0.024	0.553 *	0.674 **	0.377	-					
11	*NCDE score*	−0.131	−0.344	0.591 **	−0.154	−0.313	−0.044	0.369	0.524 *	0.356	0.644 **	-				
12	*AD score*	−0.006	−0.031	0.278	0.107	0.024	−0.046	0.248	0.289	0.469 *	0.611 **	0.293	-			
13	*RD score*	−0.127	−0.131	0.371	−0.009	−0.255	−0.097	0.670 **	0.476 *	0.392	0.852 **	0.415	0.540 *	-		
14	*Overall Visual Change Index*	−0.077	−0.349	0.310	−0.081	0.085	−0.059	0.015	0.094	−0.313	−0.032	0.023	0.050	−0.164	-	
15	*Speeding Index*	0.176	−0.502 *	0.649 **	0.129	−0.148	0.138	0.171	0.366	0.026	0.112	0.141	−0.012	0.039	0.133	-

Notes: Gender: male = 1: female = 2; AUDIT = Alcohol Use Disorders Identification Test; CUDIT-R: Cannabis Use Disorders Identification Test Revised; DDDI = Dula Dangerous Driving Index; NCED = negative cognitive/emotional driving; AD = aggressive driving; RD = risky driving. * *p* < 0.05; ** *p* < 0.01.

## Data Availability

The datasets generated during the current study are available from the corresponding author on reasonable request.
